# An integrated web medicinal materials DNA database: MMDBD (Medicinal Materials DNA Barcode Database)

**DOI:** 10.1186/1471-2164-11-402

**Published:** 2010-06-24

**Authors:** Shao-Ke Lou, Ka-Lok Wong, Ming Li, Paul Pui-Hay But, Stephen Kwok-Wing Tsui, Pang-Chui Shaw

**Affiliations:** 1 Department of Biochemistry, The Chinese University of Hong Kong, Shatin, Hong Kong, China; 2 Institute of Chinese Medicine, The Chinese University of Hong Kong, Shatin, Hong Kong, China; 3 School of Biomedical Sciences, The Chinese University of Hong Kong, Shatin, Hong Kong, China

## Abstract

**Background:**

Thousands of plants and animals possess pharmacological properties and there is an increased interest in using these materials for therapy and health maintenance. Efficacies of the application is critically dependent on the use of genuine materials. For time to time, life-threatening poisoning is found because toxic adulterant or substitute is administered. DNA barcoding provides a definitive means of authentication and for conducting molecular systematics studies. Owing to the reduced cost in DNA authentication, the volume of the DNA barcodes produced for medicinal materials is on the rise and necessitates the development of an integrated DNA database.

**Description:**

We have developed an integrated DNA barcode multimedia information platform- Medicinal Materials DNA Barcode Database (MMDBD) for data retrieval and similarity search. MMDBD contains over 1000 species of medicinal materials listed in the Chinese Pharmacopoeia and American Herbal Pharmacopoeia. MMDBD also contains useful information of the medicinal material, including resources, adulterant information, medical parts, photographs, primers used for obtaining the barcodes and key references. MMDBD can be accessed at http://www.cuhk.edu.hk/icm/mmdbd.htm.

**Conclusions:**

This work provides a centralized medicinal materials DNA barcode database and bioinformatics tools for data storage, analysis and exchange for promoting the identification of medicinal materials. MMDBD has the largest collection of DNA barcodes of medicinal materials and is a useful resource for researchers in conservation, systematic study, forensic and herbal industry.

## Background

Herbal medicine is the ancient form of pharmaceutics, which is still used by many cultures for curing diseases. With a trend of living in harmony with Nature, the use of herbal materials for treatment and health maintenance is on the rise. At present, many plant, fungal and animal species are being used for treating diseases and the Chinese Pharmacopoeia has listed 670 commonly used species [1]. Herbal medicine is a useful source of bioactive compounds, such as oils obtained from the evening primrose (*Oenothera biennis*) for treating atopic dermatitis [2], and hyperforin extracted from St. John's Wort (*Hypericum perforatum*) as an antidepressant drug [3]. Nevertheless, their efficacy is critically dependent on the use of the correct material. If toxic adulterants or substitutes are administered, life-threatening poisoning may occur. In 2002, 63 people were reported with symptoms of general malaise, nausea and vomiting after consumption of herbal tea which was inadvertently mixed with neurotoxic Japanese star anise (*Illicium anisatum*) [4]. Adulteration resulting in an epidemic of severe kidney damages caused by aristolochic acid was first reported in Belgium in 1993 [5], followed by Hong Kong and Korea [6,7] in 2004. In these cases, the concerned herbs were substituted with the nephrotoxic *Aristolochia *species. A case of misusing *Datura metel *as *Rhododendron molle *was reported in Singapore in 2008 [8]. These two species share the same Chinese herb name "Naoyanghua", but *D. metel *contains anticholinergic compound that causes confusion, dilated pupils, and absence of sweating. Traditionally, medicinal materials are identified by their organoleptic characteristics and physical properties such as shape, color, texture, and odor. However, the differences among related species or processed products are sometimes not obvious. Unique chemicals may serve as important markers for authentication, but chemical markers or profiles may be affected by the physiological and storage conditions.

With the advancement of molecular technology, DNA markers have now become a convenient means for species identification and molecular systematic study [9-11] and many DNA markers have in fact been patented for further development [12]. With the help of polymerase chain reaction (PCR), specific DNA regions can be amplified from only a small amount of samples. An unequivocal identification of a tested sample can be reached by comparing its DNA sequences against the sequence of an authentic sample. To develop a universal identification platform, The Consortium for the Barcode of Life (CBOL) proposed to set up a standardized sampling method and experimental protocol to analyze agreed-upon 'DNA barcodes' [13].

DNA barcode is a short DNA sequence of an organism, which can be used to distinguish the organism from the other species. Mitochondrial cytochrome c oxidase subunit 1 (COI) is chosen as the standard for all groups of higher animals [14,15]. For plant species, COI is not a suitable barcode because it evolves much slower than that of animals. Plant researchers examined several coding and non-coding regions, but they soon realized that a single DNA locus has limited resolving power for closely related species [16,17]. Although more laborious than the single-locus approach, it is generally agree to combine two or more barcodes to increase the successful rate [18,19]. Recently, members of the CBOL plant working group evaluated seven chloroplast genes and proposed to use *mat*K and *rbc*L as plant barcodes, based on the following criteria: easy to be amplified with a single primer pair, amenable to bidirectional sequencing with little manual editing, and high resolving power in species discrimination [20]. *rbc*L offers high universality and good discriminating power, whereas *mat*K offers higher resolution. Nevertheless, the differentiation power of these two markers may not be high in closely related plant species [21]. Also, experiences from our group and other researchers showed that chloroplast genes including *trn*H-*psb*A spacer, *trn*L-F, and nuclear regions such as internal transcribed spacer (ITS) and 5S rRNA intergenic spacer are also useful for the authentication purpose [22-24].

The Barcode of Life Data System (BOLD) is an online informatics workbench for the management, analysis and use of DNA barcodes [25], which is managed by the Canadian Center for DNA Barcoding, University of Guelph. Basically, this system utilizes COI and internal transcribed spacer (ITS) for animal and fungal identification, respectively. For plant species identification, 2-locus combination of *mat*K and *rbc*L are the default barcodes. The system also allows identification of unknown sequences up to species level provided by users.

Besides BOLD, some web-based barcode databases have been constructed to serve specific groups of organism. UNITE is an rDNA sequence database which contains 2842 ITS sequences from 1105 species of 152 genera of ectomycorrhizal fungi [26]. The main target of UNITE is to facilitate the identification of environmental samples of fungal DNA. In addition to similarity searches, UNITE has built-in maximum parsimony heuristic and neighbor joining phylogenetic tools for online analysis. All Leps Barcode of Life is a database for the identification and discovery of Lepidoptera. This database now has 448,054 barcodes from 40,907 species [27]. As insect has different morphological characters throughout its lifecycle, the DNA barcodes provide an accurate tool for the identification of the species. The Fish barcode of Life Initiative (FISH-BOL) addresses identification and natural history of various fish species through the use of COI sequence [28]. This database contains DNA barcodes, images and geospatial coordinates of examined specimens. For plant species, Genome Database for Rosaceae (GDR) is an integrated web-based relational database contains genetic markers and ESTs of the Rosaceae [29,30].

DNA barcodes are gaining popularity for authenticating medicinal materials [31-35]. Along with this trend, the 2010 edition of the Chinese Pharmacopoiea has included protocols for DNA extraction and DNA barcodes for selected medicinal materials. As a timely action, we set forth to establish the Medicinal Materials DNA Barcode Database (MMDBD) for recording the DNA barcode sequences, basic information and the key references of medicinal materials. The aims of the this database are: (1) to develop an organized and integrated web resource for DNA barcodes for medicinal species identification, (2) to collect and integrate the basic information of medicinal materials and their DNA barcodes, (3) to develop online tools and resources for sequence comparison. In this paper, we describe the structure and content of the database and reveal the database access utility and tools.

## Construction and content

### Medicinal materials information

Medicinal species listed in the Pharmacopoeia of the People's Republic of China [1], American Herbal Pharmacopoeia [36], and from prescriptions of folk medicine were chosen for including in the database. Substitutes, adulterants and closely related species were also included in the database for comparison. Currently, there are total 1259 species with 18,436 sequences available in this database. The scientific names, medicinal name, general information, and the classification of the materials in the Convention on International Trade in Endangered Species (CITES) [37] were collected. The voucher number of the samples, primer sequences and PCR conditions for generating barcode sequences are also provided as additional information. Photographs of live specimen or dried medical part of the medicinal materials were captured digitally. Live specimen images were mostly taken at the Chinese herbal garden of The Chinese University of Hong Kong (CUHK). Dried medicinal materials were provided by the Chinese Medicine Museum of the Institute of Chinese Medicine, CUHK. All images are of high resolution with 1200 × 800 pixels.

### Sequence information

#### DNA sequences selected for MMDBD

Medicinal materials include plant, animal, insect and fungal species. Considering the large number of species in the world, it is widely believed that a single set of barcodes should be adopted. Since CBOL has already chosen several DNA sequences as barcodes, undoubtedly, researchers will focus on these standard DNA regions in the future. Our primary goal is to include these DNA barcodes of medicinal materials in the MMDBD. On the other hand, many studies point out that other DNA regions are useful in species identification, and combining with the standard barcodes will increase the accuracy for differentiating closely related species. We therefore also include these "supplementary barcodes" in the MMDBD for reference. COI has been proposed as the barcode for higher animals in CBOL and BOLD [13,25]. Other mitochondrial regions, such as cytochrome b, 12S rRNA and 16S rRNA, are also proven to be useful in animal and insect species identification [38-41]. Consequently, all mitochondrial DNA sequences were included for medicinal materials originated from high animals and insects. ITS sequences have high differentiation power for the identification of fungal species [42,43], and accordingly we have selected this region for the fungal medicine. For plant materials, in addition to chloroplast DNA, nuclear DNA including ITS and 5S rRNA intergenic spacer are able to discriminate closely related plant species [23,44,45]. Also, combining two or more DNA regions may be necessary for some species. Hence, all available chloroplast DNA sequences and two nuclear gene spacers ITS and 5S rRNA intergenic spacer are included in the database. MMDBD therefore consists of barcodes proposed by CBOL and other useful DNA regions for identification of medicinal materials (Table 1).

**Table 1:** DNA barcodes and supplementary barcodes recorded in MMDBD for authenticating medicinal materials

	DNA barcode proposed by CBOL	Supplementary barcode
Plants	Chloroplast matK & rbcL	All Chloroplast DNA regions Nuclear ITS, ribosomal RNA
Animals	Mitochondrial COI	All mitochondrial DNA regions
Insects	Mitochondrial COI	All mitochondrial DNA regions
Fungi	Nuclear ITS	

#### DNA sequences extracted from public databases

INSD Seq eXtensible Markup Language (XML) files that contained DNA sequences and related information were downloaded from GenBank. Scripts were used to extract and filter sequence data from XML files, by which irrelevant sequences such as microsatellite or mRNA sequences were excluded. To keep genes without standardized names, such as 'ITS', 'ITS1', 'ITS-1' or 'ITS-2' for ITS region, multiple alternative keywords were used to keep these genes in our dataset. Then all the extracted data were imported into the MYSQL database.

#### DNA sequences generated by our groups

Our group has generated 531 DNA for 189 medicinal materials, including ITS, 5S rRNA intergenic spacer, chloroplast *trn*L, *trn*L-F and mitochondrial cytochrome b regions. Samples and specimen were collected from various localities and authenticated by experts in pharmacognosy. Total DNA was extracted and selected DNA regions were amplified by polymerase chain reaction (PCR) using universal primers [46]. The PCR products were sequenced directly or in case of unresolved sequences, the products were first cloned and sequenced. Primer flanking sites on the determined DNA sequences were removed.

### Software design and implementation

MMDBD was implemented on the relational database system MYSQL (version 5.0.45). NCBI BLAST (Basic Local Search Alignment Tool, v2.2.17) [47] was used as similarity search engine. Perl DBI module was employed to connect MYSQL, submit queries and obtain results. The MMDBD also uses the AJAX (asynchronous JavaScript and XML) technique which provides a rich and smooth user interface. Requests were posted to server-side PHP scripts and response was converted into JSON (Javascripts Object Notation) format for data interchange.

MMDBD consists of three tables which store general information, DNA sequences and references of the chosen medicinal materials (Table 2). Table "Medicinal material information" stores taxonomic, medicinal and morphological information. Field "CITESApp" records the status of endangered medicinal species in the CITES. Another field "ParmacopeiaInfor" is employed to identify all pharmacopoeia listed species in MMDBD. Table "Barcode sequence" is a sequence factory, which contains 18,436 sequences. Besides, DNA regions, voucher numbers, clone numbers, origins of samples, NCBI accession numbers and primers for PCR are assembled in this table. Foreign keys "MMId" and "RefId" are created to link with table "Medicinal material information" and "Reference", respectively. Some of the DNA sequences have been published and table "Reference" is to store the information of the articles including the author names, journal issue numbers, PubMed IDs and abstracts. It provides information for the users to trace the work that generates the DNA barcodes.

**Table 2:** Description of the three tables in MMDBD database

A) Barcode sequence	
**Field**	**Description**

SeqId	Sequence ID in MMDBD, which consists of identifier "mmdbd" and 9 digital number

DNA Region	DNA region of medicinal material

CloneNumber	Clone number

Sequence	DNA sequence of a specific region

SampleIndex	Voucher number of authentic sample

SampleOrigin	Location where sample was collected

FowardPrimer	Forward primer for PCR

ReversePrimer	Reverse primer for PCR

Source	Source of the sequence: "Y" means that it was generated by our group; "N" means that it was extracted from paper or NCBI nucleotide database

NCBIAccessNum	NCBI accession number

MMId	Foreign key of "Medicinal material information" table

RefId	Foreign key of "Reference" table

	

**B) Medicinal material information**	

**Field**	**Description**

MMId	Medicinal material ID

SpeciesName	Species name of medicinal material

VariantName	Variant name of medicinal material

Family	Family name of medicinal material

MMName	Medicinal name of medicinal material

MMType	Type of medicinal material: "P" for plant, "A" for animal, "I" for insect, and "F" for fungus

MedicalPart	Medical part of medicinal material

CITESApp	Appendices of CITES listed species

ParmacopeiaInfor	Listed species in Chinese Pharmacopoeia or American Herbal Pharmacopoeia

Adulterant	Adulterant of medicinal material

MMPhoto	File name of medicinal material image

NChs	Stroke number of medicinal material name in simplified Chinese

	

**C) Reference**	

**Field**	**Description**

RefId	Reference ID

PMID	PubMed Unique Identifier (PMID) from PubMed

Title	Title of reference

Author	Author name of reference

Journal	Journal title, publication volume, date and page number

Abstract	Abstract of reference

Language	Language used

## Utility and discussion

### Database access and tools

**Figure 1:**
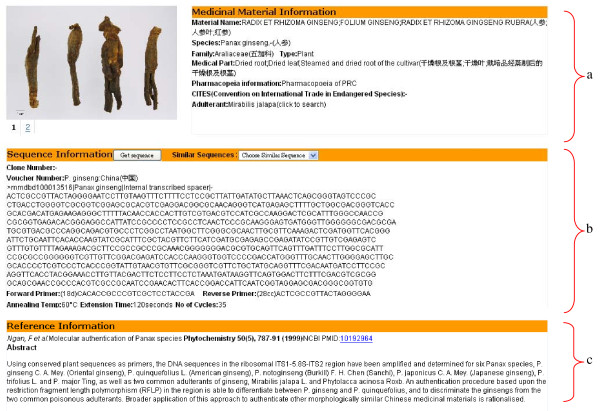
**Information page for medicinal materials**. Information page shows general information of medicinal materials, including **(a) **herb name, species name, family name, medical part, pharmacopoeia information, status in CITES, adulterant, **(b) **DNA sequence and **(c) **key reference.

MMDBD provides a simple web-based interface to retrieve barcode information (Fig. 1). The URL is: http://www.cuhk.edu.hk/icm/mmdbd.htm.

MMDBD has three major functions: database query by text-based interface, sequence similarity search, and data submission. Researchers can enrich the database by contributing their DNA barcode sequences to MMDBD. For data submission, we have provided a template file in EXCEL format, which enables users to upload batches of sequences and information via email. The administrator will then check the data quality before incorporating them to the database.

### Database search interface

#### Search view

**Figure 2:**
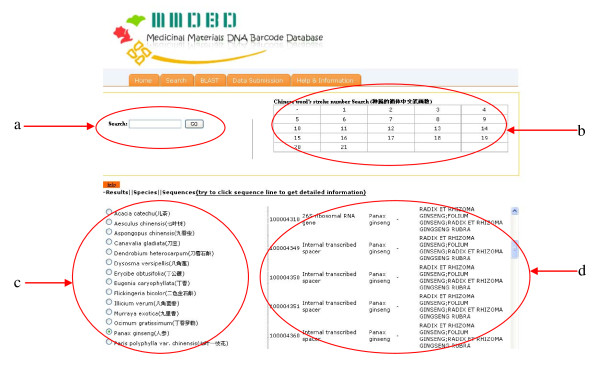
**Result page for medicinal material search**. There are two search methods: **(a) **keyword search and **(b) **Chinese character stroke number search. (**c**) The search result is displayed at the lower left block. By clicking the button before the species name, (**d**) the list of available DNA sequences of the chosen species is shown at the lower right block. User can get the desired information of the medicinal materials by following the hyperlinks provided.

There are two search methods: keywords search and Chinese character stroke number search (Fig. 2). In the former, user can enter a single word or phrase to search the herb name, species name, family name and references of the medicinal materials. The keywords are passed to server in UTF-8 encoding which supports both simplified and traditional Chinese characters. Another search method is by means of stroke number of Chinese species name. Stroke number table was created according to the stroke number of the first Chinese character of the species name. User can get the desired information of medicinal materials by following the hyperlinks in the table.

#### Sequence similarity search

**Figure 3:**
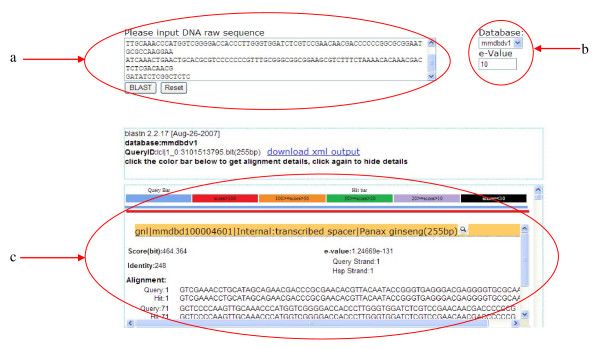
**Sequence similarity search result using built-in BLAST**. MMDBD allows users to conduct **(a) **homology searches between their sequences of interest and the data in the database **(b) **with adjustable E-value. Result page displays **(c) **sequence homology and links to the information page.

MMDBD utilizes BLASTN algorithm for sequence similarity search. This function allows users to conduct homology searches between their sequences of interest and the data in the database (Fig. 3). The input sequence should be in raw sequence format and the length must be between 10 and 2000 bases. The word size in BLASTN is preset to 7 in order to improve algorithm search sensitivity for short sequence. User is also able to adjust the E-value to further optimize the searching results. The BLAST output page displays the sequence homology in a rich interface in which user can go to the details page of the target barcode sequence by clicking the color bar links.

#### Future development

The database has already covered 66.5% and 84.5% of the medicinal materials listed in the Chinese Pharmacopoeia and the American Herbal Pharmacopoeia, respectively. It provides an open forum for further addition of medicinal materials barcodes. In addition, efforts will focus on improving the tools and functionality of the web interface such as an advanced search site with options for search or display categories.

## Conclusions

The MMDBD is initiated to support the DNA barcoding initiative, which includes numerous important medicinal materials. MMDBD contains DNA barcode sequences, basic information and references of the medicinal materials. The integrated database provides users with easy access and retrieval of the data and web tools for sequence comparison. MMDBD will play a timely and important role for the authentication and quality control of medicinal materials and benefit the herbal industry. It will also be useful to investigators for conservation, forensic and systematic analysis.

## Availability and requirements

The MMDBD is publicly available and can be accessed at http://www.cuhk.edu.hk/icm/mmdbd.htm.

## Authors' contributions

SKL performed web interface design, rational-relationship database construction and manuscript writing. KLW wrote the XSLT for converting eXtensible Markup Language (XML) data, collected and input data into the database and wrote the manuscript. ML collected and input the data into the database. PCS, SKWT and PPHB conceived and supervised the project. All authors read and approved the final manuscript.

## References

[B1] The Pharmacopoeia Editorial Committee Pharmacopoeia of the People's republic of China 2005 Shanghai: Chemical Technology Press

[B2] Stonemetz D Donnelly GF A review of the clinical efficacy of evening primrose Holistic Nursing Practice 2008 22 Philadelphia, PA: Lippincott Williams & Wilkins, Inc 171 174 18453897 10.1097/01.HNP.0000318026.45527.07

[B3] Medina MA Martínez-Poveda B Amores-Sánchez MI Quesada AR Hyperforin: more than an antidepressant bioactive compound? Life Sci 2006 79 105 111 10.1016/j.lfs.2005.12.027 16438991

[B4] Johanns ES Van der Kolk LE Van Gemert HM Sijben AE Peters PW De Vries I An epidemic of epileptic seizures after consumption of herbal tea Ned Tijdschr Geneeskd 2002 146 813 816 12014242

[B5] Vanherweghem JL Depierreux M Tielemans C Abramowicz D Dratwa M Jadoul M Richard C Vandervelde D Verbeelen D Vanhaelen-Fastre R Rapidly progressive interstitial renal fibrosis in young women: association with slimming regimen including Chinese herbs Lancet 1993 341 387 391 10.1016/0140-6736(93)92984-2 8094166

[B6] Lo SH Mo KL Wong KS Poon SP Chan CK Lai CK Chan A Aristolochic acid nephropathy complicating a patient with focal segmental glomerulosclerosis Nephrol Dial Transplant 2004 19 1913 1915 10.1093/ndt/gfh159 15199198

[B7] Lee S Lee T Lee B Choi H Yang M Ihm CG Kim M Fanconi's syndrome and subsequent progressive renal failure caused by a Chinese herb containing aristolochic acid Nephrology 2004 9 126 129 10.1111/j.1440-1797.2003.00232.x 15189173

[B8] Phua DH Cham G Seow E Two instances of Chinese herbal medicine poisoning in Singapore Singapore Med J 2008 49 e131 3 18465037

[B9] Kitaoka F Kakiuchi N Long CF Itoga M Mitsue A Mouri C Mikage M Molecular characterization of *Akebia *plants and the derived traditional herbal medicine Biol Pharm Bull 2009 32 665 670 10.1248/bpb.32.665 19336902

[B10] Holderegger H Abbott RJ Phylogeography of the arctic-alpine *Saxifraga oppositifolia *(Saxifragaceae) and some related taxa based on cpDNA and ITS Am J Bot 2003 90 931 936 10.3732/ajb.90.6.931 21659189

[B11] Kress WJ Wurdack KJ Zimmer EA Weigt LA Janzen DH Use of DNA barcodes to identify flowering plants Proc Natl Acad Sci USA 2005 102 8369 8374 10.1073/pnas.0503123102 15928076 PMC1142120

[B12] Shaw PC Wong KL Chan AWK Wong WC But PPH Patent applications for using DNA technologies to authenticate medicinal herbal material Chin Med 2009 4 21 10.1186/1749-8546-4-21 19930671 PMC2791102

[B13] Consortium for the Barcode of Life http://www.barcoding.si.edu/

[B14] Dawnay N Ogden R McEwing R Carvalho GR Thorpe RS Validation of the barcoding gene CO1 for use in forensic genetic species identification Forensic Sci Int 2007 173 1 6 10.1016/j.forsciint.2006.09.013 17300895

[B15] Yoo HS Eah JY Kim JS Kim YJ Min MS Paek WK Lee H Kim CB DNA barcoding Korean birds Mol Cells 2006 22 323 327 17202861

[B16] Kress WJ Erickson DL A two-locus global DNA barcode for land plants: the coding rbcL gene complements the non-coding trnH-psbA spacer region PLoS One 2007 2 e508 10.1371/journal.pone.0000508 17551588 PMC1876818

[B17] Fazekas AJ Burgess KS Kesanakurti PR Graham SW Newmaster SG Husband BC Percy DM Hajibabaei M Barrett SCH Multiple Multilocus DNA Barcodes from the Plastid Genome Discriminate Plant Species Equally Well PLoS One 2008 3 e2802 10.1371/journal.pone.0002802 18665273 PMC2475660

[B18] Kress WJ Erickson DL Jones FA Swenson NG Perez R Sanjur O Bermingham E Plant DNA barcodes and a community phylogeny of a tropical forest dynamics plot in Panama Proc Natl Acad Sci USA 2009 106 18621 6 10.1073/pnas.0909820106 19841276 PMC2763884

[B19] Newmaster SG Ragupathy S Ethnobotany genomics - discovery and innovation in a new era of exploratory research J Ethnobiol Ethnomed 2010 6 2 10.1186/1746-4269-6-2 20102622 PMC2828978

[B20] CBOL Plant Working Group A DNA barcode for land plants Proc Natl Acad Sci USA 2009 106 12794 12797 10.1073/pnas.0905845106 19666622 PMC2722355

[B21] Cameron KM Chase MW Whitten WM Kores PJ Jarrell DC Albert VA Yukawa T Hills HG Goldman DH A phylogenetic analysis of the Orchidaceae: evidence from *rbc*L nucleotide sequences Am J Bot 1999 86 208 224 10.2307/2656938 21680360

[B22] Poon WS Shaw PC Simmons MP But PPH Congruence of molecular, morphological, and biochemical profiles in Rutaceae: a cladistic analysis of the subfamilies Rutoideae and Toddalioideae Systematic Botany 2007 32 837 846

[B23] Sun Y Fung KP Leung PC Shaw PC A phylogenetic analysis of *Epimedium *(Berberidaceae) based on nuclear ribosomal DNA sequences Mol Phylogenet Evol 2005 35 287 291 10.1016/j.ympev.2004.12.014 15737598

[B24] Ferri G Alù M Corradini B Beduschi G Forensic botany: species identification of botanical trace evidence using a multigene barcoding approach Int J Legal Med 2009 123 395 401 10.1007/s00414-009-0356-5 19504263

[B25] Ratnasingham S Hebert PDN BOLD: The Barcode of Life Data System Mol Ecol Notes 2007 7 355 364 10.1111/j.1471-8286.2007.01678.x 18784790 PMC1890991

[B26] Kõljalg U Larsson KH Abarenkov K Nilsson RH Alexander IJ Eberhardt U Erland S Høiland K Kjøller R Larsson E Pennanen T Sen R Taylor AF Tedersoo L Vrålstad T Ursing BM UNITE: a database providing web-based methods for the molecular identification of ectomycorrhizal fungi New Phytologist 2005 166 1063 1068 10.1111/j.1469-8137.2005.01376.x 15869663

[B27] Lepidoptera Barcode of Life http://www.lepbarcoding.org/

[B28] The Fish Barcode of Life Initiative (FISH-BOL) http://www.fishbol.org/

[B29] Jung S Staton M Lee T Blenda A Svancara R Abbott A Main D GDR (Genome Database for Rosaceae): integrated web-database for Rosaceae genomics and genetics data Nucl Acids Res 2008 36 D1034 D1040 10.1093/nar/gkm803 17932055 PMC2238863

[B30] Genome Database for Rosaceae http://www.bioinfo.wsu.edu/gdr/

[B31] Song J Yao H Li Y Li X Lin Y Liu C Han J Xie C Chen S Authentication of the family Polygonaceae in Chinese pharmacopoeia by DNA barcoding technique J Ethnopharmacol 2009 124 434 439 10.1016/j.jep.2009.05.042 19505556

[B32] Zhang J Wang J Xia T Zhou S DNA barcoding: species delimitation in tree peonies Sci China C Life Sci 2009 52 568 578 10.1007/s11427-009-0069-5 19557335

[B33] Yao H Song JY Ma XY Liu C Li Y Xu HX Han JP Duan LS Chen SL Identification of *Dendrobium *species by a candidate DNA barcode sequence: the chloroplast *psb*A-*trn*H intergenic region Planta Med 2009 75 667 669 10.1055/s-0029-1185385 19235685

[B34] Chen S Yao H Han J Liu C Song J Shi L Zhu Y Ma X Gao T Pang X Luo K Li Y Li X Jia X Lin Y Leon C Validation of the ITS2 region as a novel DNA barcode for identifying medicinal plant species PLoS One 2010 7 e8613 10.1371/journal.pone.0008613 PMC2799520 20062805

[B35] Asahina H Shinozaki J Masuda K Morimitsu Y Satake M Identification of medicinal *Dendrobium *species by phylogenetic analyses using *matK *and *rbcL *sequences J Nat Med 2010 64 133 8 10.1007/s11418-009-0379-8 20140532

[B36] American herbal pharmacopoeia http://www.herbal-ahp.org/index.html

[B37] Convention on International Trade in Endangered Species of Wild Fauna and Flora http://www.cites.org/10.1159/000459796 712806

[B38] Wong KL Wang J But PPH Shaw PC Application of ctyochrome b DNA sequences for the authentication of endangered snake species Forensic Sci Int 2004 139 49 55 10.1016/j.forsciint.2003.09.015 14687773

[B39] Gupta AR Patra RC Das DK Gupta PK Swarup D Saini M Sequence characterization and polymerase chain reaction-restriction fragment length polymorphism of the mitochondrial DNA 12S rRNA gene provides a method for species identification of Indian deer Mitochondrial DNA 2008 19 394 400 19462513 10.1080/19401730802351251

[B40] Barr NB Copeland RS De Meyer M Masiga D Kibogo HG Billah MK Osir E Wharton RA McPheron BA Molecular diagnostics of economically important *Ceratitis *fruit fly species (Diptera: Tephritidae) in Africa using PCR and RFLP analyses Bull Entomol Res 2006 96 505 521 17092362

[B41] Ferri G Alù M Corradini B Licata M Beduschi G Species identification through DNA "barcodes" Genet Test Mol Biomarkers 2009 13 421 6 10.1089/gtmb.2008.0144 19405876

[B42] Crouch JA Clarke BB Hillman BI What is the value of ITS sequence data in *Colletotrichum *systematics and species diagnosis? A case study using the falcate-spored graminicolous *Colletotrichum *group Mycologia 2009 101 648 656 10.3852/08-231 19750944

[B43] Zeng JS De Hoog GS *Exophiala spinifera *and its allies: diagnostics from morphology to DNA barcoding Med Mycol 2008 46 193 208 10.1080/13693780701799217 18404547

[B44] Ruzicka J Lukas B Merza L Göhler I Abel G Popp M Novak J Identification of *Verbena officinalis *based on ITS sequence analysis and RAPD-derived molecular markers Planta Med 2009 75 1271 1276 10.1055/s-0029-1185535 19350481

[B45] Maruyama T Kamakura H Miyai M Komatsu K Kawasaki T Fujita M Shimada H Yamamoto Y Shibata T Goda Y Authentication of the traditional medicinal plant *Eleutherococcus senticosus *by DNA and chemical analyses Planta Med 2008 74 787 789 10.1055/s-2008-1074537 18500683

[B46] Chen F Chan HY Wong KL Wang J Yu MT But PP Shaw PC Authentication of *Saussurea lappa*, an endangered medicinal material, by ITS DNA and 5S rRNA sequencing Planta Med 2008 74 889 892 10.1055/s-2008-1074551 18537077

[B47] Zhang Z Schwartz S Wagner L Miller W A greedy algorithm for aligning DNA sequences J Comput Biol 2000 7 203 214 10.1089/10665270050081478 10890397

